# Unimolecular GLP-1/APJ receptor co-agonism improves metabolism and dyslipidaemia as well as normalising hepatic triglyceride and aminotransferase elevation in high-fat-fed mice

**DOI:** 10.1007/s00125-026-06863-7

**Published:** 2026-09-18

**Authors:** Ananyaa Sridhar, Ethan S. Palmer, Sarah L. Craig, Neil Tanday, Finbarr P. M. O’Harte, Nigel Irwin

**Affiliations:** Centre for Diabetes, School of Biomedical Sciences, Ulster University, Coleraine, UK

**Keywords:** Apelin, APJ receptor, Body weight, GLP-1, Gluconeogenesis, Metabolism

## Abstract

**Aims/hypothesis:**

The aim of this study was to determine the applicability of dual activation of glucagon-like peptide-1 (GLP-1) and apelin (APJ) receptors as a next-generation therapeutic option for obesity and diabetes.

**Methods:**

A fully characterised unimolecular dual GLP-1/APJ receptor agonist, namely exendin–linker–apelin (ELA), as well as its acylated long-acting equivalent, ELA-Lys^12^(γGluPal), were administered once daily to high-fat-fed (HFF) mice for 28 days, with liraglutide used as a positive control. Effects on appetite and body weight, as well as glucose, pyruvate and insulin tolerance, circulating lipids, liver transaminases and triglyceride content, indirect calorimetry measurements and pancreatic morphology were then examined.

**Results:**

ELA, and particularly ELA-Lys^12^(γGluPal), reduced food intake, circulating glucose and HbA_1c_ concentrations relative to saline-treated HFF control mice. ELA-Lys^12^(γGluPal) substantially reduced blood glucose concentrations during glucose and pyruvate tolerance tests. These positive effects were largely independent of alterations in circulating insulin. However, circulating triglyceride and LDL-cholesterol concentrations were lowered, accompanied by complete normalisation of hepatic triglyceride and aminotransferase levels in all GLP-1/apelin hybrid-treated HFF mice. Assessment of indirect calorimetry demonstrated restoration of metabolic flexibility in mice treated with ELA-Lys^12^(γGluPal), despite reductions in energy expenditure. Notably, GLP-1/apelin hybrid treatment did not affect locomotor activity, whereas liraglutide treatment was associated with marked reductions in physical activity. Histological assessment of pancreatic islets revealed that all treatment modalities induced a marked enhancement of beta cell proliferative frequency, and accompanying suppression of apoptotic rates, without major alterations in islet or beta cell areas.

**Conclusions/interpretation:**

Chronic administration of ELA, and especially ELA-Lys^12^(γGluPal), delivered pronounced metabolic improvements in HFF mice, with the clearest differences from liraglutide treatment evident in circulating lipids and hepatic biomarkers.

**Graphical Abstract:**

**Figure:**
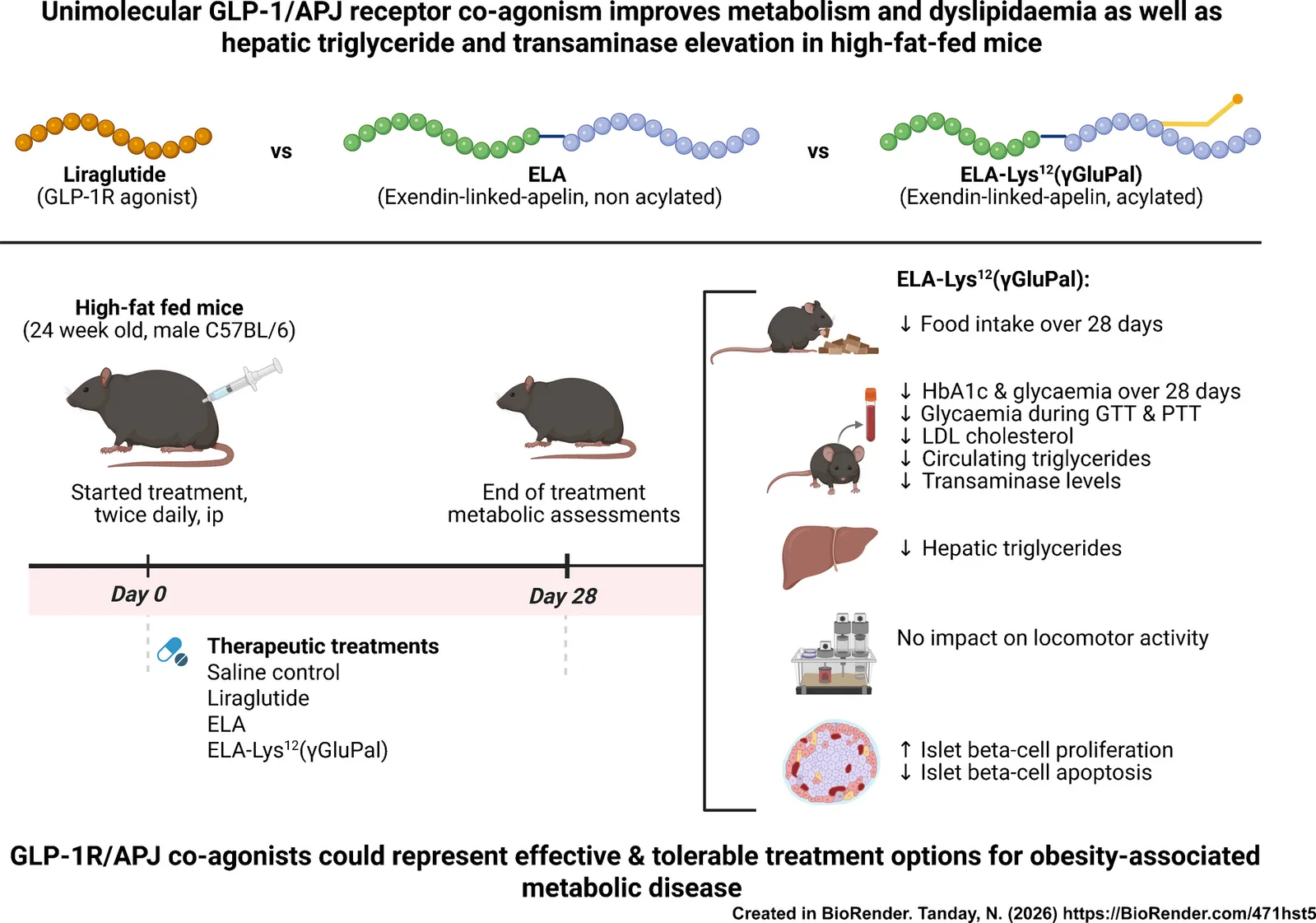

**Supplementary Information:**

The online version contains peer-reviewed but unedited supplementary material available at https://doi.org/10.1007/s00125-026-06863-7.

**Figure:**
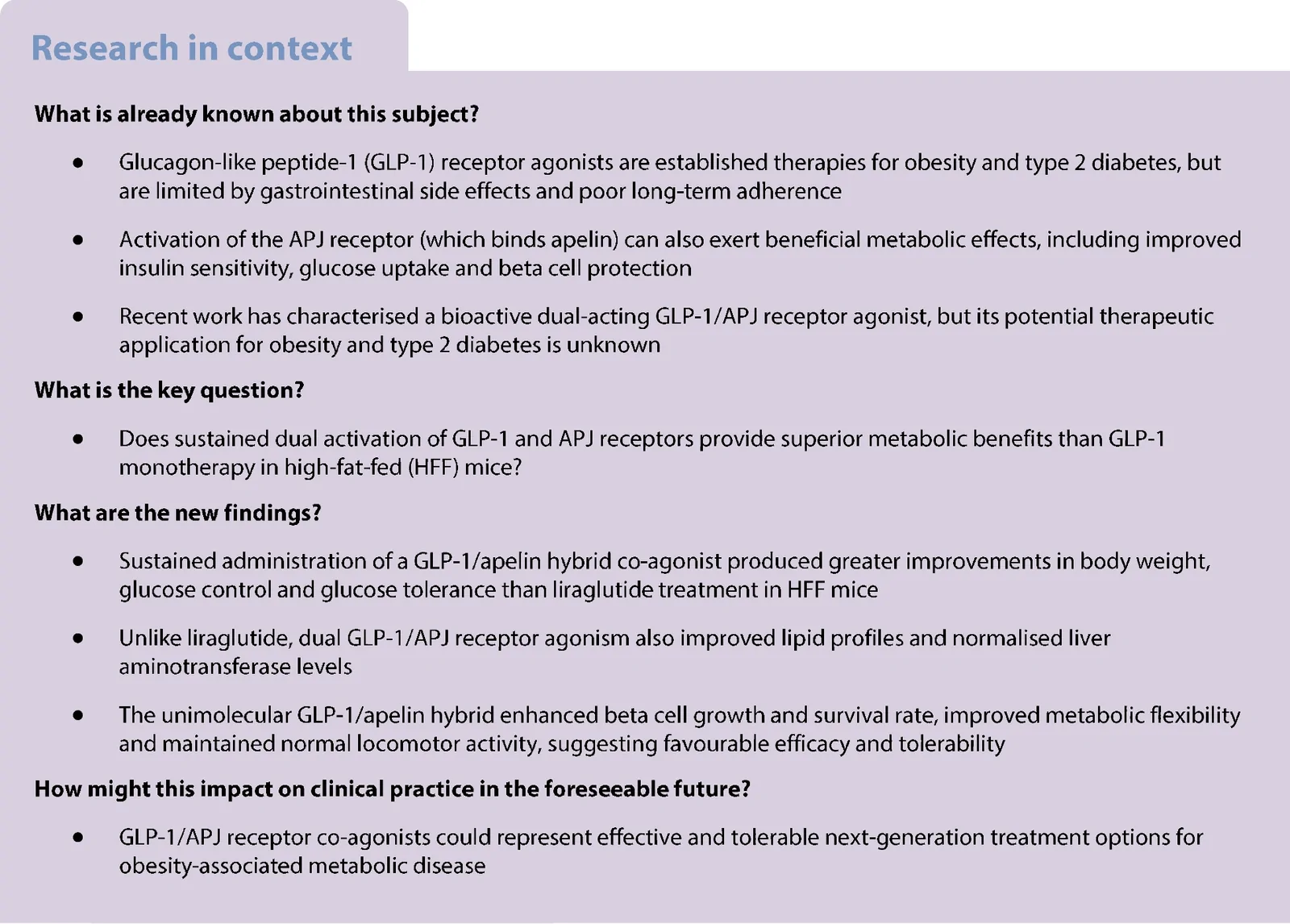

## Introduction

Obesity and type 2 diabetes mellitus are linked to chronic insulin resistance, with an additional central pathological hallmark of type 2 diabetes being progressive deterioration of pancreatic beta cell function [1, 2]. In this context, glucagon-like peptide-1 (GLP-1) therapies have improved patient outcomes, representing an option that effectively improves glucose management while also encouraging weight loss [3]. In particular, the dual-acting drug tirzepatide, which co-activates receptors for GLP-1 and its sister incretin hormone glucose-dependent insulinotropic polypeptide, demonstrates notable efficacy [4]. However, as with the GLP-1 monotherapy treatments [5], tirzepatide is associated with gastrointestinal side effects leading to high discontinuation rates [6], linked to reduced long-term treatment adherence or lack of sustainable efficacy [7]. Therefore, there is still notable enthusiasm for the generation of more effective and tolerable incretin-like therapies [8].

To address this, we have recently characterised a set of chimeric GLP-1/apelin hybrid peptides that promote prominent appetite suppression without induction of obvious malaise, as well as enhancing beta cell survival rate and glucose regulation [9]. It is well recognised that GLP-1 activates Gs-coupled cAMP and PKA-dependent cell signalling pathways to provoke metabolic benefits [10], while the adipokine apelin binds to the angiotensin II receptor-like protein J (APJ) receptor, a Gi/o- and Gq/11-linked G-protein-coupled receptor that predominantly engages the PI3K/Akt, MAPK/ERK and phospholipase C intracellular signalling cascades [11]. Thus, GLP-1 and apelin receptors engage partially distinct intracellular signalling pathways, raising the possibility that unimolecular co-agonists may broaden the metabolic actions achieved with GLP-1 receptor agonism alone. Similar to GLP-1 [12], apelin has been shown to exert pancreatic beta cell protective effects and promote glucose lowering, and to enhance glucose uptake as well as improving insulin sensitivity and overall cardiovascular function [13]. Indeed, it has recently been suggested that APJ receptor activation may act synergistically to enhance the cardioprotective efficacy of GLP-1 [14]. Given the well-documented cardiovascular benefits of GLP-1 drugs in diabetes [15], together with possible reductions of circulating apelin in cardiovascular disease [16], this proposed synergistic action could evolve into a crucial therapeutic benefit for GLP-1/apelin hybrid peptides.

In our initial work with GLP-1/apelin hybrid peptides, we generated exendin–linker–apelin (ELA) as a selective GLP-1/APJ receptor dual agonist, as well as related long-acting acylated analogues, with ELA-Lys^12^(γGluPal) emerging as a promising lead [9]. Both ELA and ELA-Lys^12^(γGluPal) activated GLP-1 and APJ receptor-mediated cell signalling pathways and exerted prolonged biological actions following single peptide injections in rodents [9]. To advance the translatability of these findings, the primary objective of the present study was to evaluate the metabolic, lipid, hepatic and pancreatic islet benefits of 28 days of administration of ELA and ELA-Lys^12^(γGluPal) in high-fat-fed (HFF) mice. Importantly, HFF mice mirror many of the abnormalities of obesity/diabetes in humans, including insulin resistance, elevated blood glucose, hepatic steatosis and altered lipid profiles [12]. We hypothesised that chronic treatment with GLP-1/apelin hybrid peptides will produce greater and more sustained improvements in metabolic control and cardiovascular-related outcomes than GLP-1 receptor agonism alone, while potentially reducing treatment-limiting side effects.

## Methods

### Peptides

Peptides were purchased from Synpeptide (Shanghai, China) at 95% purity. Characterisation was corroborated in-house using HPLC and MALDI-TOF MS analysis, as previously described [17].

### Animals and treatment schedules

Further details of experimental conditions are provided in the electronic supplementary material (ESM Methods). Briefly, male mice (Envigo, Loughborough, UK) maintained on a high-fat diet (45% fat; Research Diets, New Brunswick, USA) for 14 weeks were randomly allocated into groups (*n*=8) that received once-daily i.p. injections (at 10:00 hours) of either saline vehicle (0.9% NaCl), liraglutide, ELA or ELA-Lys^12^(γGluPal) (each at 25 nmol/kg body weight), as applicable, for 28 days. Lean mice received once-daily saline injections (0.9% NaCl, i.p.) throughout.

### Metabolic analyses

At the end of the treatment period, aspects of indirect calorimetry were measured [18]. Following this, a GTT (18 mmol/kg, i.p.), insulin sensitivity test (5 U/kg bovine insulin, i.p.) and pyruvate tolerance test (2 g/kg sodium pyruvate; i.p.) were performed. End of study analyses included measurement of total body fat mass in addition to collection of plasma for determination of the plasma lipid profile, circulating aspartate aminotransferase (AST), alanine aminotransferase (ALT) and insulin concentrations. See ESM Methods for further details of experimental conditions.

### Biochemical analyses

Blood glucose levels were recorded using a handheld Ascencia Contour glucometer (Bayer Healthcare, Newbury, UK) and plasma insulin was assessed by in-house radioimmunoassay [19]. Circulating lipids and liver enzymes were measured using a Hitachi 912 automated analyser (Boehringer Ingelheim, Mannheim, Germany). Hepatic triglycerides were quantified by colorimetric analysis (EEA028 triglyceride kit, Invitrogen/ThermoFisher Scientific, Altrincham, UK). See ESM Methods for further details of experimental conditions.

### Pancreatic islet immunohistochemistry

Islet morphology, as well as measurement of beta cell proliferation and apoptosis and related image analysis, were conducted as described previously [20, 21]. See ESM Methods for further details of experimental conditions. Slides were imaged using a fluorescence microscope (model BX51; Olympus, Southend-on-Sea, UK) fitted with FITC (488 nm) and TRITC (594 nm) filters and an Olympus XM10 camera system.

### Statistical analysis

Statistical analysis was performed using GraphPad PRISM software version 10.3.0 (GraphPad, San Diego, CA, USA). Values are expressed as means ± SEM. Comparative analyses between groups were conducted using one-way ANOVA or two-way ANOVA with Bonferroni post hoc test. For repeated-measures analyses, sphericity was evaluated and the Geisser–Greenhouse correction was applied where necessary. All statistical tests were two-tailed, and the difference between groups was considered significant if at a *p* value <0.05.

## Results

### ELA, and particularly ELA-Lys^12^(γGluPal), decrease percentage body weight gain and improve glucose control in HFF mice

**Fig. 1:**
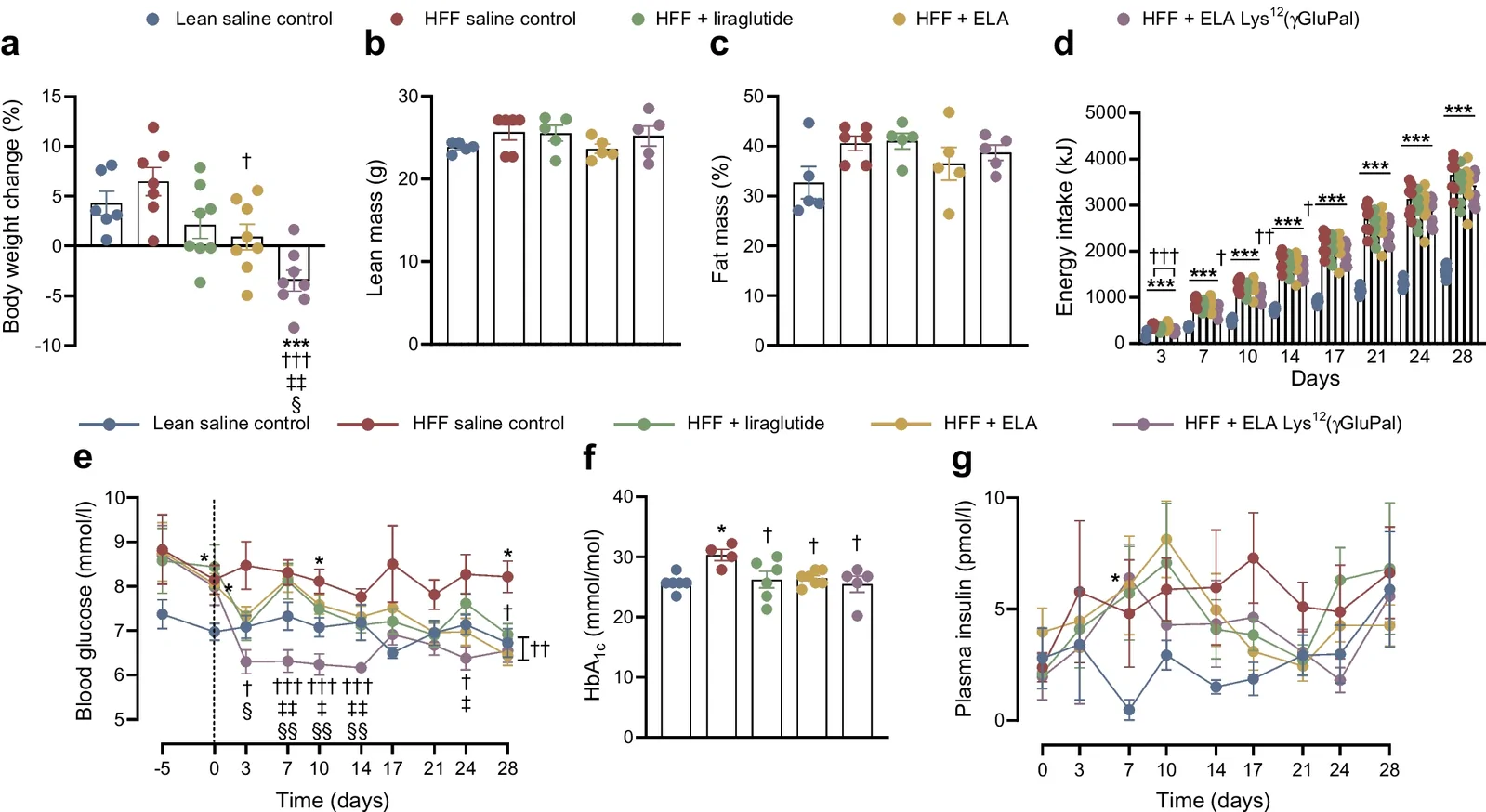
Effects of sustained dual GLP-1 and APJ receptor agonism on (**a**) body weight change and (**b**, **c**) body composition, (**d**) energy intake, (**e**, **f**) glucose control and (**g**) circulating insulin in HFF mice. (**a**–**c**, **f**) Parameters were assessed after 28 days of treatment with once-daily saline in lean and HFF mice, as well as once-daily treatment with liraglutide, ELA or ELA-Lys^12^(γGluPal) (each at 25 nmol/kg body weight) in HFF mice. (**d**, **e**, **g**) Parameters were assessed at regular intervals during the treatment period in HFF mice. Data are presented as means ± SEM (*n*=4–8 independent observations per group). *p<0.05, ***p<0.001 compared with lean control; ^†^*p*<0.05, ^††^*p*<0.01, ^†††^*p*<0.001 compared with HFF control, ^‡^*p*<0.05, ^‡‡^*p*<0.01 compared with liraglutide; ^§^*p*<0.05, ^§§^*p*<0.01 compared with ELA

Both ELA and ELA-Lys^12^(γGluPal) significantly reduced percentage body weight gain following 28 days of once-daily treatment, when compared with saline-treated HFF control mice (*p*<0.05–*p*<0.001; Fig. 1a). Notably, ELA-Lys^12^(γGluPal) encouraged greater reductions in percentage body weight gain when compared with both liraglutide- and ELA-treated HFF mice, as well as lean control mice (*p*<0.05–*p*<0.001; Fig. 1a). Interestingly, there were no differences in lean and fat body mass between the groups of mice, suggesting a uniform modulation of body composition by all treatment interventions (Fig. 1b, c). As expected, energy intake was elevated throughout in HFF mice, compared with mice maintained on a standard rodent diet (*p*<0.001; Fig. 1d). In addition, HFF mice treated with ELA-Lys^12^(γGluPal) presented with significantly reduced cumulative energy intake relative to HFF control mice during the earlier phase of treatment, specifically on days 3, 7, 10 and 14 (*p*<0.05–*p*<0.001; Fig. 1d). Energy intake remained numerically reduced in these mice throughout the remainder of the study, but this effect was non-significant from day 17 onwards (Fig. 1d). Remarkably, ELA-Lys^12^(γGluPal) intervention also led to sustained and consistent reductions in blood glucose levels when compared with saline-, ELA- and liraglutide-treated HFF mice (*p*<0.05–*p*<0.001; Fig. 1e), with ELA and liraglutide therapy modestly decreasing circulating glucose only when compared with HFF control mice (*p*<0.05–*p*<0.01; Fig. 1e). HbA_1c_, as assessed on day 28, was elevated in HFF mice when compared with lean control mice (*p*<0.05; Fig. 1f), but was normalised by all peptide treatments (Fig. 1f). There was no significant difference in non-fasting plasma insulin concentrations between the groups of mice, barring a small transient increase on day 7 in mice treated with ELA-Lys^12^(γGluPal) when compared with healthy lean control mice (Fig. 1g).

### ELA-Lys^12^(γGluPal) improves glucose tolerance as well as decreasing hepatic gluconeogenesis in HFF mice

**Fig. 2:**
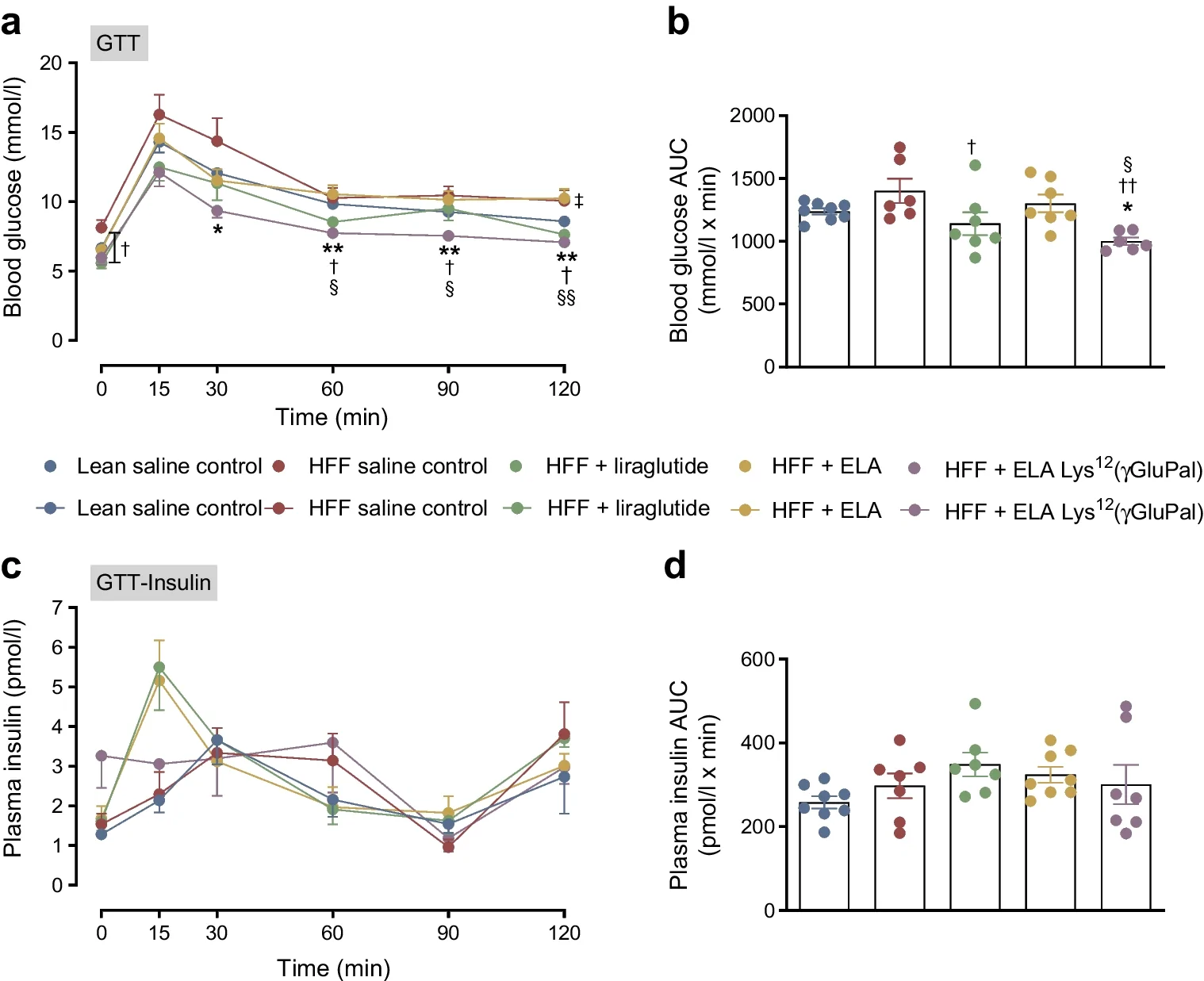
Effects of sustained dual GLP-1 and APJ receptor agonism on glucose tolerance and glucose-stimulated insulin secretion in HFF mice. All parameters were assessed after 28 days of treatment with once-daily saline in lean and HFF mice, as well as once-daily treatment with liraglutide, ELA or ELA-Lys^12^(γGluPal) (each at 25 nmol/kg body weight) in HFF mice. (**a**, **b**) Blood glucose and (**c**, **d**) plasma insulin were measured prior to and after administration of glucose alone (18 mmol/kg body weight) at *t*=0 min in mice fasted overnight (16 h). Data are expressed as means ± SEM (*n*=6–8 independent observations per group). *p<0.05, **p<0.01 compared with lean control; ^†^*p*<0.05, ^††^*p*<0.01 compared with HFF control, ^‡^*p*<0.05 compared with liraglutide; ^§^*p*<0.05, ^§§^*p*<0.01 compared with ELA

At the end of the 28-day treatment period, an i.p. GTT was performed to assess dynamic glucose handling (Fig. 2a). At baseline, mice treated with liraglutide, ELA or ELA-Lys^12^(γGluPal) exhibited significantly lower fasting blood glucose when compared with saline-treated HFF control mice (*p*<0.05; Fig. 2a). Following glucose administration, mice treated with ELA-Lys^12^(γGluPal) presented with superior and persistent overall plasma glucose control, including lowered individual and 0–120 min AUC glucose values when compared with saline-treated lean and HFF control mice, as well as ELA-treated HFF mice (*p*<0.05–*p*<0.01; Fig. 2a, b). Liraglutide-treated HFF mice also exhibited improved 0–120 min AUC glucose levels when compared with saline-treated HFF control mice (*p*<0.05; Fig. 2b). Associated glucose-induced plasma insulin concentrations were not noticeably different between groups of mice, although prior to the glucose challenge at 0 min mice treated with ELA-Lys^12^(γGluPal) did seem to have elevated, albeit non-significantly, plasma insulin concentrations when compared with HFF control mice (0.019 ± 0.005 vs 0.009 ± 0.001 ng/ml, respectively; Fig. 2c, d).

**Fig. 3:**
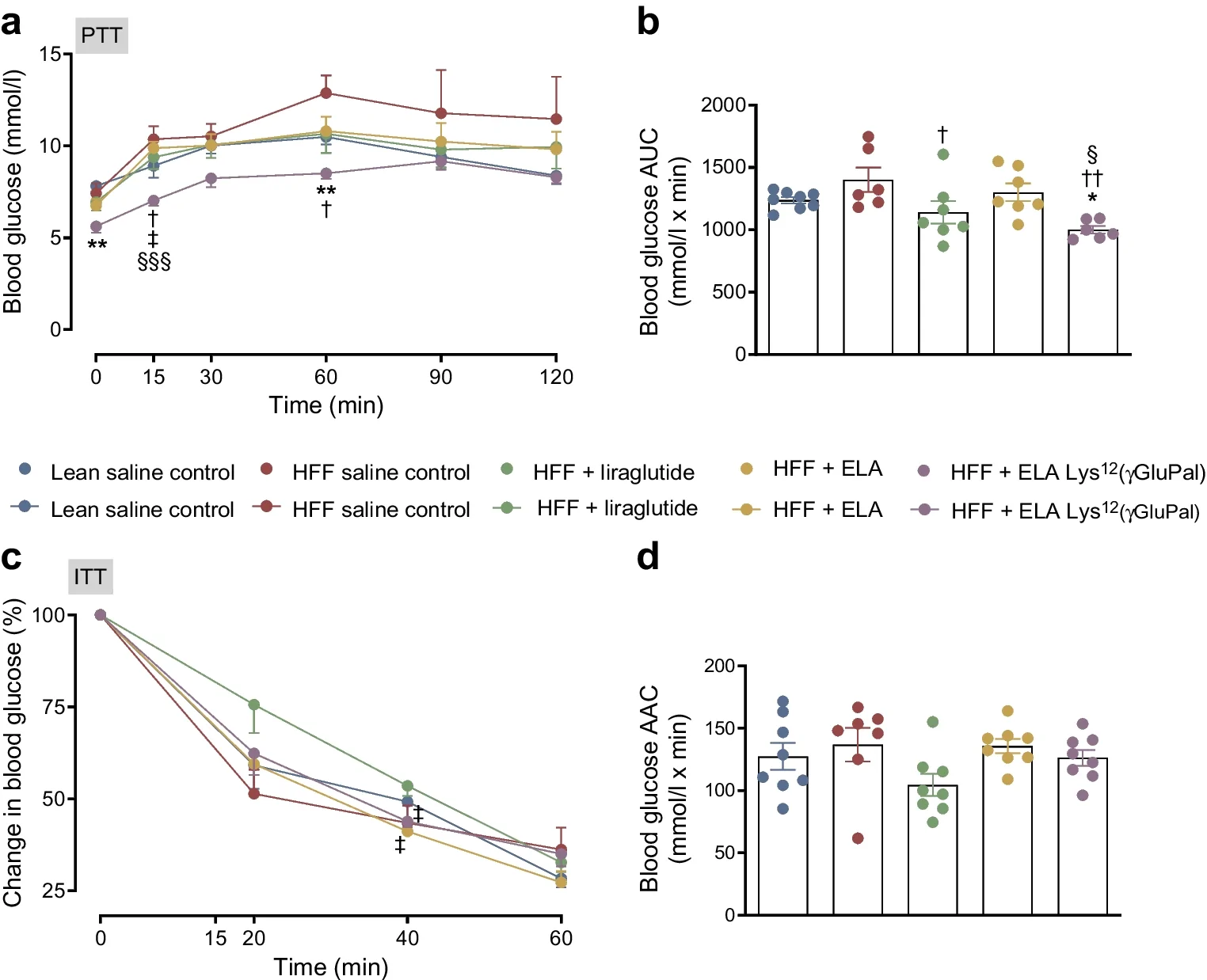
Effects of sustained dual GLP-1 and APJ receptor agonism on (**a**, **b**) pyruvate tolerance and (**c**, **d**) insulin sensitivity in HFF mice. All parameters were assessed after 28 days of treatment with once-daily saline in lean and HFF mice, as well as once-daily treatment with liraglutide, ELA or ELA-Lys^12^(γGluPal) (each at 25 nmol/kg body weight) in HFF mice. (**a**, **b**) Blood glucose was measured prior to and after administration of sodium pyruvate (2 g/kg body weight) at *t*=0 min in mice fasted overnight (16 h). (**c**, **d**) Blood glucose was measured prior to and after administration of insulin (5 U/kg body weight) at *t*=0 min in non-fasted mice. Data are expressed as means ± SEM (*n*=6–8 independent observations per group). *p<0.05, **p<0.01 compared with lean control; ^†^*p*<0.05, ^††^*p*<0.01 compared with HFF control, ^‡^*p*<0.05 compared with liraglutide; ^§^*p*<0.05, ^§§§^*p*<0.001 compared with ELA. AAC, area above the curve

To assess hepatic gluconeogenic capacity, a pyruvate tolerance test was conducted (Fig. 3a). Notably, mice treated with ELA-Lys^12^(γGluPal) had significantly reduced blood glucose concentrations for at least one observation point when compared with all other groups of mice (*p*<0.05–*p*<0.001, Fig. 3a). Analysis of 0–120 min AUC values demonstrated a significant reduction of overall blood glucose levels in HFF mice treated with ELA-Lys^12^(γGluPal) when compared with saline-treated lean and HFF control mice, as well as ELA-treated HFF mice (*p*<0.05–*p*<0.01; Fig. 3b). The hypoglycaemic action of exogenous insulin administration was also examined, with ELA and ELA-Lys^12^(γGluPal) treatment leading to modest, but observable, reductions in blood glucose when compared with liraglutide-treated HFF mice (*p*<0.05; Fig. 3c). However, there was no obvious difference in 0–60 min area above the curve (AAC) blood glucose values between all groups of mice following exogenous insulin injection (Fig. 3d).

### ELA-Lys^12^(γGluPal) differentially modulates energy expenditure, respiratory exchange ratio and locomotor activity in HFF mice

**Fig. 4:**
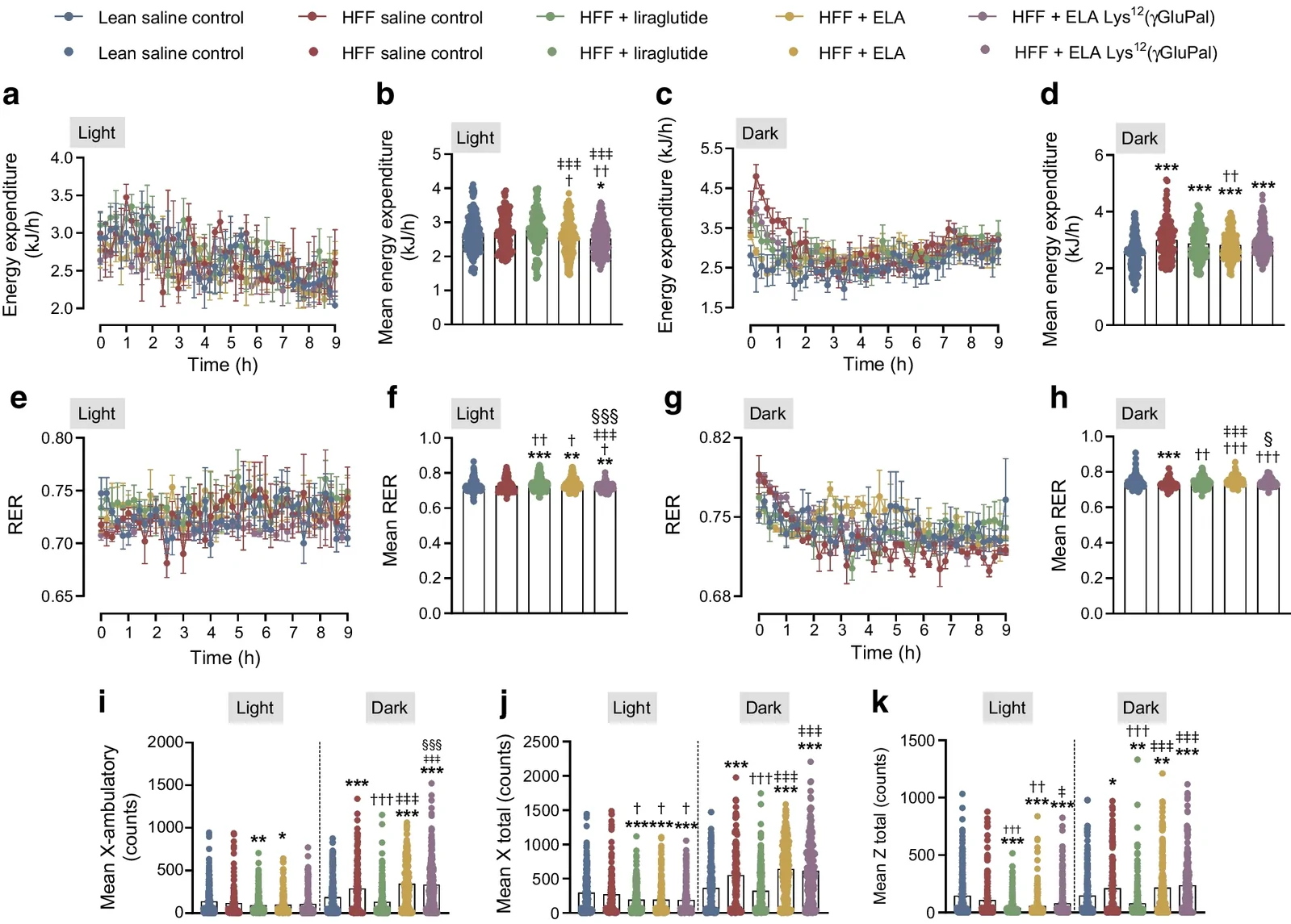
Effects of sustained dual GLP-1 and APJ receptor agonism on (**a**–**d**) energy expenditure, (**e**–**h**) RER and (**i**–**k**) locomotor activity in HFF mice. All parameters were assessed after 28 days of treatment with once-daily saline in lean and HFF mice, as well as once-daily treatment with liraglutide, ELA or ELA-Lys^12^(γGluPal) (each at 25 nmol/kg body weight) in HFF mice. (**a**–**d**) Energy expenditure and (**e**–**h**) RER during the (**a**, **b**, **e**, **f**) light and (**c**, **d**, **g**, **h**) dark phases were assessed. (**i**–**k**) Activity counts on the (**i**, **j**) *x*-axis (X-ambulatory) and (**k**) *z*-axis (Z counts) were recorded at 60 s intervals, and overall incremental data are presented. Data represent means ± SEM (*n*=8 per group; dot plots display all serial measurements collected from each animal over the observation period). *p<0.05, **p<0.01, ***p<0.001 compared with lean control; ^†^*p*<0.05, ^††^*p*<0.01,^†††^*p*<0.001 compared with HFF control, ^‡^*p*<0.05, ^‡‡‡^*p*<0.001 compared with liraglutide; ^§^*p*<0.05, ^§§§^*p*<0.001 compared with ELA

During the light phase, energy expenditure was comparable between saline-treated lean and HFF control mice, as well as liraglutide-treated HFF mice, but significantly reduced in the HFF mice treated with ELA or ELA-Lys^12^(γGluPal) (*p*<0.05–*p*<0.001; Fig. 4a, b). Notably, HFF mice treated with ELA-Lys^12^(γGluPal) had decreased energy expenditure when compared with saline-treated lean and HFF control mice, as well as liraglutide-treated HFF mice during the light phase (*p*<0.05–*p*<0.001; Fig. 4a, b). During the dark phase, all HFF mice displayed elevated energy expenditure compared with lean mice (*p*<0.001; Fig. 4c, d), with 28 days of ELA treatment reducing energy expenditure relative to HFF controls (*p*<0.01; Fig. 4d). High-fat feeding did not alter the respiratory exchange ratio (RER) during the light phase (Fig. 4e, f). However, liraglutide and ELA treatment significantly increased RER when compared with HFF control mice (*p*<0.05–*p*<0.01; Fig. 4e, f), whereas treatment with ELA-Lys^12^(γGluPal) reduced RER (*p*<0.05–*p*<0.001; Fig. 4f). During the dark phase, RER was significantly lower in saline-treated HFF control mice when compared with lean control mice (*p*<0.001; Fig. 4g, h). All treatments reversed this effect, with ELA mice exhibiting elevated RER when compared with HFF mice treated with liraglutide and ELA-Lys^12^(γGluPal) (*p*<0.05–*p*<0.001; Fig. 4g, h). Analysis of locomotor activity revealed reductions in X-ambulatory counts in liraglutide- and ELA-treated HFF mice during the light phase, when compared with lean control mice (*p*<0.05–*p*<0.01; Fig. 4i). During the dark phase, X-ambulatory counts were prominently elevated in all HFF mice, except those treated with liraglutide (*p*<0.001; Fig. 4i). In addition, HFF mice treated with ELA or ELA-Lys^12^(γGluPal) exhibited further increases in X-ambulatory activity during the dark phase in comparison with HFF control mice (*p*<0.001; Fig. 4i). Mean X-ambulatory counts were unaltered by high-fat feeding during the light phase, but were reduced by treatment with liraglutide, ELA or ELA-Lys^12^(γGluPal) (*p*<0.05–*p*<0.001; Fig. 4j). During the dark phase, X-ambulatory counts were increased in all HFF mice, expect those treated with liraglutide (*p*<0.001; Fig. 4j), with ELA or ELA-Lys^12^(γGluPal) treatment inducing increases in this parameter when compared with liraglutide (*p*<0.001; Fig. 4j). Vertical movement, as assessed by Z counts, was reduced by all treatment interventions throughout the light phase compared with lean and HFF control mice (*p*<0.05–*p*<0.001; Fig. 4k). Interestingly, during the dark phase, Z counts were significantly increased (*p*<0.05–*p*<0.001; Fig. 4k) in all HFF mice when compared with lean control mice, except in those mice treated with liraglutide, where Z counts were actually reduced when compared with healthy lean control mice (*p*<0.01; Fig. 4k).

### ELA and ELA-Lys^12^(γGluPal) improve the plasma lipid profile and reduce hepatic triglyceride and aminotransferase levels in HFF mice

**Fig. 5:**
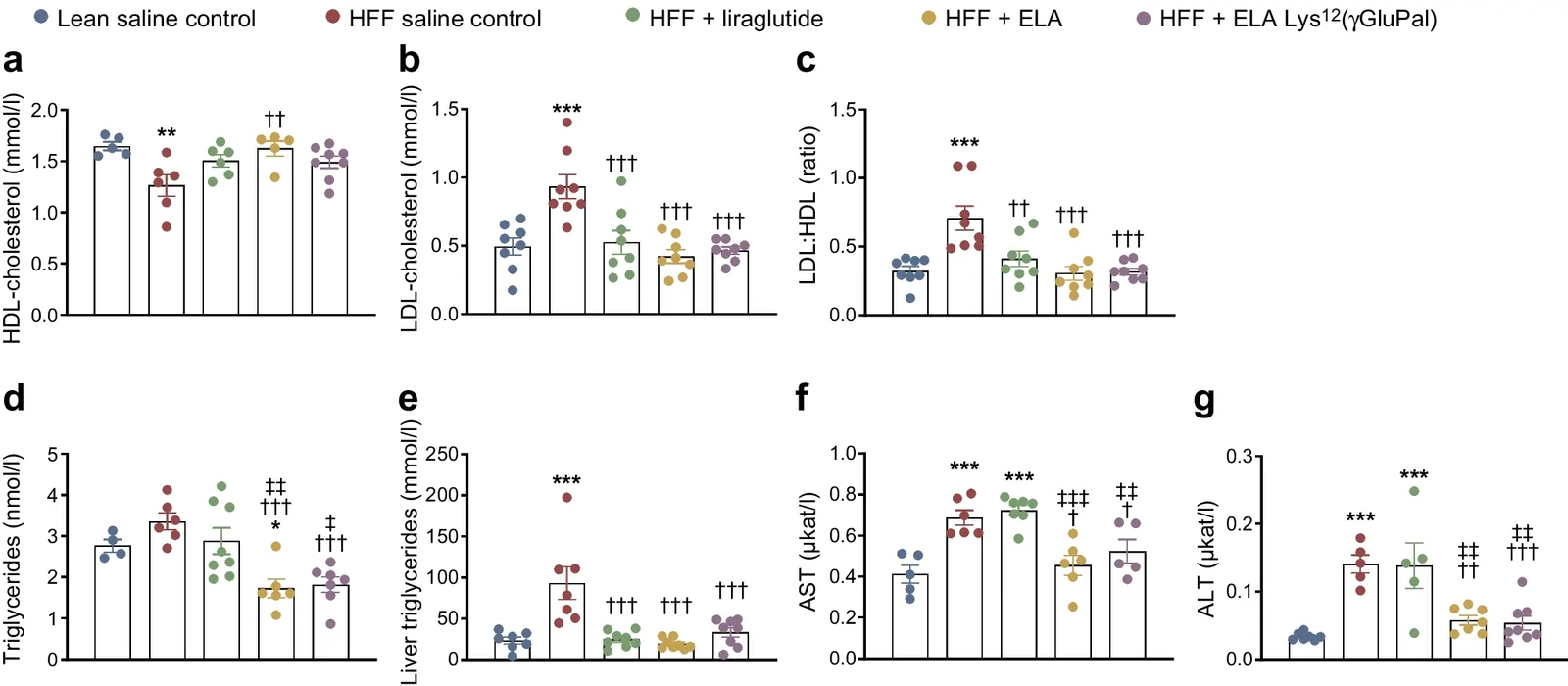
Effects of sustained dual GLP-1 and APJ receptor agonism on (**a**–**d**) the plasma lipid profile, (**e**) hepatic triglyceride content and (**f**, **g**) circulating AST and ALT in HFF mice. All parameters were assessed after 28 days of treatment with once-daily saline in lean and HFF mice, as well as once-daily treatment with liraglutide, ELA or ELA-Lys^12^(γGluPal) (each at 25 nmol/kg body weight) in HFF mice. The plasma lipid profile and aminotransferase enzymes were measured in plasma collected at the end of the study. Hepatic triglycerides were assessed by colorimetric analysis after ethanol extraction. Data are presented as means ± SEM (*n*=4–8 independent observations per group). *p<0.05, **p<0.01, ***p<0.001 compared with lean control; ^†^*p*<0.05, ^††^*p*<0.01, ^†††^*p*<0.001 compared with HFF control, ^‡^*p*<0.05, ^‡‡^*p*<0.01, ^‡‡‡^*p*<0.001 compared with liraglutide

High-fat feeding markedly reduced HDL-cholesterol levels, but all treatments returned HDL-cholesterol concentrations to lean control levels, with ELA-treated HFF mice having increased HDL-cholesterol compared with their liraglutide-treated counterparts (*p*<0.01; Fig. 5a). LDL-cholesterol was substantially elevated in saline-treated HFF control mice (*p*<0.001; Fig. 5b) and restored to normal levels by all three treatment paradigms. Consistent with these changes, the LDL:HDL ratio was increased in saline-treated HFF control mice, but was similar to that in lean control mice in all HFF treatment groups (*p*<0.05–*p*<0.001; Fig. 5c). Plasma triglyceride concentrations were reduced in both the ELA and ELA-Lys^12^(γGluPal) HFF groups when compared with saline-treated lean and HFF control mice, as well as liraglutide-treated HFF mice (*p*<0.05–0.001; Fig. 5d). Furthermore, saline-treated HFF control mice exhibited elevated hepatic triglyceride content (*p*<0.001; Fig. 5e). However, all treatment interventions reduced liver triglycerides, returning these to similar levels to those observed in lean control mice (Fig. 5e). AST and ALT were markedly elevated in HFF control mice (*p*<0.001; Fig. 5f, g), with liraglutide treatment unable to reverse this negative dietary-induced effect. However, ELA and ELA-Lys^12^(γGluPal) treatment significantly reduced circulating ALT and AST concentrations when compared with saline- and liraglutide-treated HFF mice (*p*<0.05–*p*<0.001), returning levels similar to those observed in lean control mice (Fig. 5f, g).

### ELA and ELA-Lys^12^(γGluPal) enhance proliferation and limit apoptosis of pancreatic islet beta cells in HFF mice

**Fig. 6:**
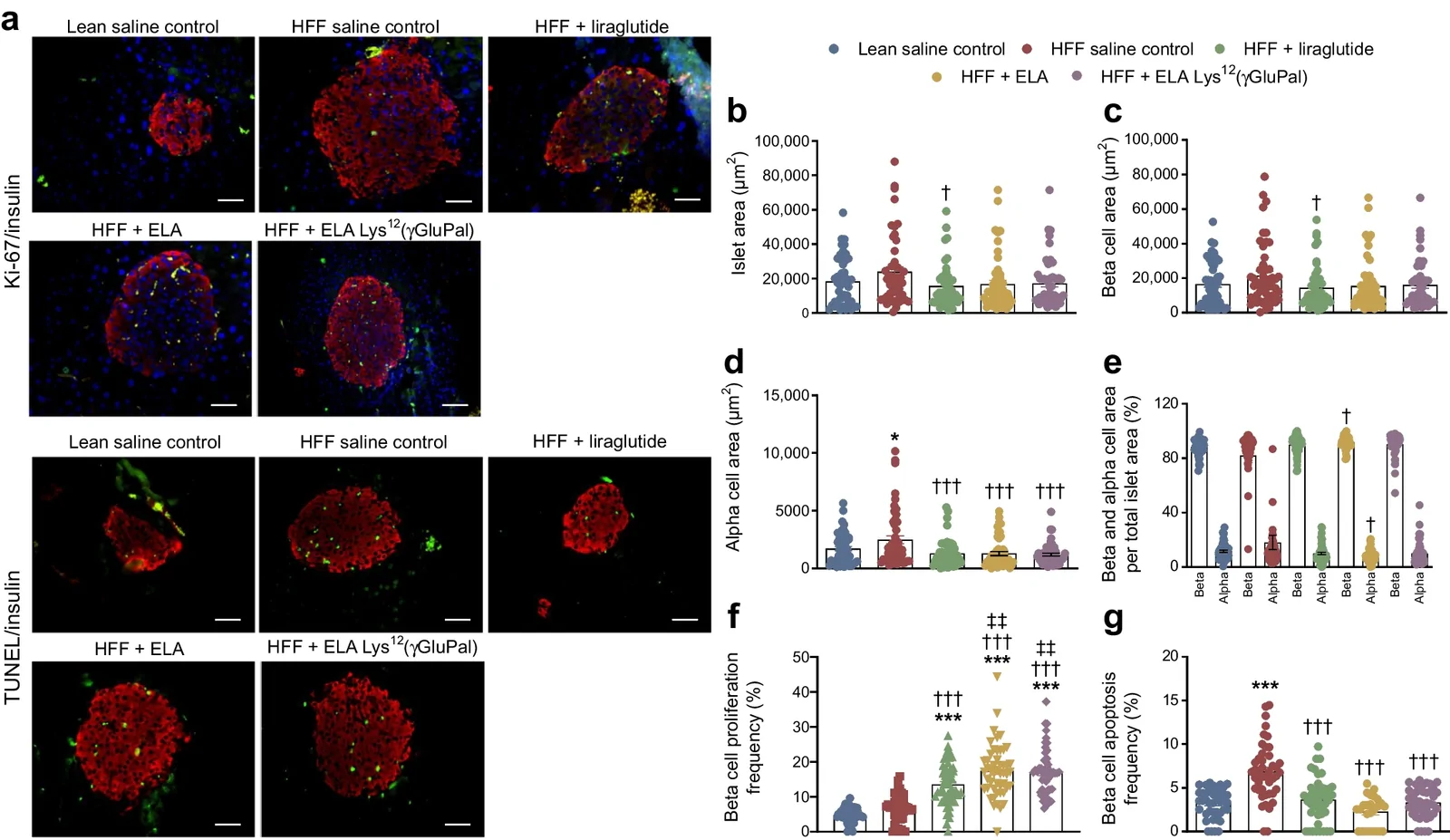
Effects of sustained dual GLP-1 and APJ receptor agonism on (**a**–**e**) pancreatic islet architecture as well as (**f**) beta cell proliferation rate and (**g**) beta cell apoptosis rate in HFF mice. All parameters were assessed after 28 days of treatment with once-daily saline in lean and HFF mice, as well as once-daily treatment with liraglutide, ELA or ELA-Lys^12^(γGluPal) (each at 25 nmol/kg body weight) in HFF mice. (**a**) Representative immunofluorescent images of pancreatic islets stained for insulin (red), Ki-67 or using TUNEL (green) and DAPI (blue) from all groups of mice. Scale bar=50 μm. (**b**–**e**) Quantitative morphometric analyses of pancreatic islets parameters included measurement of (**b**) total islet area, (**c**) beta cell area, (**d**) alpha cell area and (**e**) percentage beta and alpha cell area relative to total islet area. (**f**, **g**) Quantification of (**f**) beta cell proliferation and (**g**) beta cell apoptosis frequency using Ki-67 and TUNEL staining, respectively. Data are expressed as means ± SEM (*n*=8 per group; dot plots display all measurements from 60–80 islets per group). *p<0.05, ***p<0.001 compared with lean control; ^†^*p*<0.05, ^†††^*p*<0.001 compared with HFF control; ^‡‡^*p*<0.01 compared with liraglutide

Representative immunofluorescent images of pancreatic islets from each group of mice stained for insulin and Ki-67 or assayed by TUNEL, as appropriate, are shown in Fig. 6a. Quantitative morphometric analysis revealed that total islet and beta cell areas were modestly, but significantly, reduced in liraglutide-treated mice when compared with saline-treated HFF control mice (*p*<0.05; Fig. 6b, c). The other treatment interventions, namely ELA and ELA-Lys^12^(γGluPal), did not impact islet or beta cell areas (Fig. 6b, c). The alpha cell area was elevated in HFF control mice (*p*<0.05), but markedly reduced by liraglutide, ELA and ELA-Lys^12^(γGluPal) treatments to levels comparable with those of healthy lean control mice (*p*<0.001; Fig. 6d). When expressed as a percentage of total islet area, ELA treatment increased percentage beta cell area, while concomitantly reducing the percentage of alpha cell area (*p*<0.05; Fig. 6e), with all other treatments having no obvious impact on this morphometric analysis (Fig. 6e). Interestingly, beta cell proliferation frequency was increased in HFF mice treated with liraglutide, ELA or ELA-Lys^12^(γGluPal) when compared with both saline-treated lean mice and HFF control mice (*p*<0.01–*p*<0.001; Fig. 6f). Moreover, ELA and ELA-Lys^12^(γGluPal) induced significantly higher beta cell proliferation frequency than liraglutide (*p*<0.01 Fig. 6f). In addition, high-fat feeding significantly increased beta cell apoptosis frequency, but this was fully reversed by all treatment interventions (*p*<0.001; Fig. 6g).

## Discussion

Sustained 28-day GLP-1/APJ receptor co-agonism gave rise to prominent and integrated metabolic effects in HFF mice. While several endpoints were similarly improved by liraglutide and the ELA hybrid treatments, the clearest between-treatment differences were observed for plasma lipid profile, liver aminotransferases and selected glucose tolerance measures, particularly with ELA-Lys^12^(γGluPal). This may imply that pharmacokinetic optimisation is critical for maximising the therapeutic efficacy of GLP-1/apelin hybrid drugs [9]. It is also worth noting that the magnitude of liraglutide-mediated benefits on body composition and circulating glucose and insulin was more modest than might be expected. This probably reflects study-specific aspects such as model severity, treatment duration and experimental design, as well as awareness of the increased liraglutide dose requirement for obesity as opposed to type 2 diabetes in the clinical setting [22]. Accordingly, comparisons between liraglutide and the ELA hybrid peptides should be interpreted in the context of the mouse model employed. An additional study limitation is that the degree of separation between saline-treated lean and HFF control mice was somewhat modest for certain dynamic metabolic measures, consistent with an intermediate diet-induced metabolic phenotype. Nonetheless, the current HFF model did present clear elevations of body weight, hepatic triglycerides, HbA_1c_ and AST/ALT concentrations, as well as overt dyslipidaemia, confirming the negative impact of the dietary intervention.

High-fat feeding in rodents generally leads to a rapid elevation of body weight, coupled with a more progressive impairment of glucose metabolism linked to chronic beta cell stress [23]. In the current study, ELA, and particularly ELA-Lys^12^(γGluPal), reduced fasting and postprandial blood glucose levels, as well as restricting weight gain. Recent debate has focused on the impact of GLP-1-based therapies on preservation of lean mass, especially muscle [24], with various targeted therapies being developed to combat this [25]. However, in good agreement with a recent study in mice and humans [26], as well as a network meta-analysis [27], we show that the percentage of lean mass relative to total body weight was unaffected by liraglutide, or either of the GLP-1/apelin hybrid molecules, despite clear reductions of percentage body weight gain with ELA-Lys^12^(γGluPal). In any case, lean mass is an imperfect surrogate for skeletal muscle function [28], with apelin considered to reverse age-associated sarcopenia [29]. There was also an associated decline of energy intake with ELA-Lys^12^(γGluPal) treatment, but this effect was modest and diminished over time, suggesting that reduced food intake alone is unlikely to fully explain the observed decrease in percentage body weight gain.

To further probe potential mechanisms, an assessment of energy metabolism was performed at the end of the treatment period, meaning that such observations only represent a single metabolic snapshot, and underlying mechanisms require further targeted investigation, although even small alterations in metabolic rate would be therapeutically significant, provided they were sustained throughout the entire study. Both ELA and ELA-Lys^12^(γGluPal) reduced energy expenditure, particularly during the light phase, which may partly reflect reduced locomotor activity, as observed previously [30]. While this may initially seem counterintuitive, changes in energy expenditure need to be viewed within the context of reduced weight gain and improved glucose and hepatic outcomes, as well as enhanced locomotor activity. RER measurements further indicated that dual GLP-1/APJ receptor activation alters systemic fuel utilisation. High-fat feeding typically reduces metabolic flexibility and promotes preferential lipid oxidation [31], with liraglutide and GLP-1/apelin hybrid treatments increasing RER during the active dark phase, indicating enhanced carbohydrate utilisation [32]. In accordance with these findings, both chronic apelin and GLP-1 treatment have previously been shown to decrease RER in diet-induced obesity [33, 34]. More interestingly, HFF mice treated with the most effective intervention, namely ELA-Lys^12^(γGluPal), presented with diurnal variation in RER that could be linked to circadian rhythms of macronutrient metabolism and augmented metabolic flexibility [35], which merit further investigation.

There was a sharp contrast in nocturnal locomotor activity between the liraglutide-treated HFF mice and those treated with the GLP-1/apelin hybrids, with liraglutide significantly reducing activity, consistent with reports that GLP-1R agonists suppress spontaneous activity [36]. Given that the dark phase is associated with active feeding in rodents, reduced movement may be linked to the well-characterised induction of malaise by GLP-1 mimetics [5]. Previous observations confirm that a single injection of 25 nmol/kg ELA in rodents leads to prolonged anorectic effects without such adverse outcomes [9]. This may represent a significant therapeutic advantage for ELA compounds over related dual-acting peptide therapies such as tirzepatide, as GLP-1 receptor engagement appears to drive gastrointestinal side effects with these drugs [37], a complication that is notably absent with ELA treatment despite its GLP-1 component [9]. Nevertheless, direct comparisons with clinically advanced multi-agonists, such as tirzepatide or retatrutide, were beyond the scope of the present study. Lack of obvious malaise also helps verify that reduced energy expenditure following ELA or ELA-Lys^12^(γGluPal) treatment was not linked to impaired metabolic health. More fundamentally, reduced locomotor activity could potentially limit long-term metabolic benefits in treatment of obesity.

Glucose tolerance testing revealed marked improvements in glucose homeostasis following ELA-Lys^12^(γGluPal) treatment that did not appear to be directly linked to a meaningful enhancement of beta cell responsiveness. However, it should be recognised that experimental limitations precluded a full evaluation of first-phase insulin responses, and we were unable to assess insulin secretory responsiveness in isolated islets. Although GLP-1 is a recognised insulin secretagogue [38], APJ receptor activation is linked with augmented glucose uptake in peripheral tissues [39]. It is interesting that fasting insulin concentrations were slightly elevated in mice treated with ELA-Lys^12^(γGluPal), as this can inversely correlate with insulin sensitivity [2]. That said, the hypoglycaemic effect of exogenous insulin was not appreciably altered by any of the treatment interventions. However, this lack of effect may be linked to the relatively indirect method used to assess whole-body insulin sensitivity, and activation of inherent negative feedback mechanisms to prevent the onset of hypoglycaemia [40]. In that respect, euglycaemic–hyperinsulinaemic clamp studies would have provided a more reliable readout of insulin action, but are beyond the scope of the current study. Nonetheless, high-fat diets promote increased hepatic gluconeogenesis and triglyceride accumulation that contribute to hyperglycaemia and insulin resistance [41], with liraglutide, and particularly ELA-Lys^12^(γGluPal), clearly decreasing hepatic gluconeogenesis as established through assessment of pyruvate tolerance. This finding is in harmony with prior observations of apelin- and GLP-1-mediated improvements in hepatic insulin sensitisation [42, 43]. Hepatic triglyceride accumulation is a defining feature of obesity-associated steatosis, closely linked to excessive gluconeogenic flux [44], and was largely reversed by liraglutide and GLP-1/apelin hybrid peptide treatment. Coupled with the restoration of liver aminotransferase enzymes and plasma lipid homeostasis, there is a clear indication that the observed metabolic benefits in HFF mice treated with ELA-Lys^12^(γGluPal) are fundamentally underpinned by enhanced hepatic function. Given the close association between steatotic liver disease and type 2 diabetes, therapies that simultaneously improve glucose levels and hepatic biomarkers are of particular clinical interest [44].

Despite the absence of clear changes in circulating or glucose-stimulated insulin concentrations, we performed an in-depth profiling of pancreatic islet histology given the documented benefits of sustained GLP-1 and APJ receptor activation on islet morphology [45, 46] and the established detrimental impact of high-fat feeding on beta cell health [47]. Notably, assessment of circulating glucose and insulin levels over the 28-day treatment period was conducted in the freely fed state, rendering these data susceptible to meal-dependent fluctuations. Furthermore, while GLP-1 and APJ receptor desensitisation are theoretical considerations for ELA and ELA-Lys^12^(γGluPal), this seems unlikely. For example, sustained administration of GLP-1 therapies rarely induces clinically significant receptor downregulation [12], and apelin-13, the precursor apelin component of both ELA hybrid peptides, promotes more rapid receptor recycling than other apelin isoforms [48].

All treatment modalities, and especially GLP-1/apelin hybrid peptides, enhanced beta cell proliferation and restrained beta cell apoptosis. However, this did not translate to an obvious augmentation of islet morphology or beta cell area, barring a marginal increase of beta to alpha cell ratio in ELA-treated mice. The explanation for this is not obvious, and may relate to the lack of dynamic analysis across the entire observation period, with observations being made only upon study termination. In addition, pancreatic islet cell plasticity has recently been shown to play a significant role in the maintenance of normal islet morphology and function, as well as in the pathogenesis of obesity-driven forms of diabetes [49], and could also be an important factor in these observations. Furthermore, both GLP-1 and apelin receptor activation have been shown to modulate islet cell lineage and functional cellular identity [50, 51], and this process merits further investigation in appropriate lineage tracing models. Consistent with this, the apelinergic system has been implicated in beta cell mass expansion during pregnancy [52], a process that has recently been linked to alterations in islet cell plasticity [53].

One limitation of the current study that should be acknowledged is that isolation of the specific contributions of GLP-1 and APJ receptor activation to the observed phenotype was not explored. Inclusion of a dedicated apelin agonist comparator, together with liraglutide, would probably have helped define the relative contribution of APJ vs GLP-1 receptor activation to the metabolic benefits induced by the ELA peptides. Intriguingly, we have previously demonstrated that sustained independent chronic APJ receptor agonism in HFF mice produced several comparable metabolic benefits to those of the ELA peptides, including improved glucose homeostasis, together with favourable effects on circulating lipid and hepatic biomarkers [18]. The current data reinforce the concept that APJ receptor activation contributes to metabolic regulation in diet-induced obesity [9]. While model and treatment regimen differences may make it difficult to tease out the exact therapeutic benefits of ELA peptides over single apelin or GLP-1 agonists [3, 18], the current study verifies that dual GLP-1 and APJ receptor signalling possesses clear experimental promise for metabolic disease, building on earlier conceptual proposals [9, 12, 13].

### Conclusions

The present findings, combined with previous observations [9], provide compelling evidence to support the development of long-acting GLP-1/APJ receptor co-agonists as a next-generation strategy for obesity-associated metabolic disease. The ability of ELA, and especially ELA-Lys^12^(γGluPal), to improve multiple metabolic domains simultaneously, coupled with favourable tolerability, distinguishes the current approach from other GLP-1-centric therapies, including the recently approved dual GLP-1/glucose-dependent insulinotropic polypeptide receptor activator tirzepatide [4]. In this context, ELA-Lys^12^(γGluPal) offers a differentiated strategy that may help overcome some of the current limitations associated with existing incretin-based therapies.

## Supplementary Information

Below is the link to the electronic supplementary material.

ESM Methods (PDF 132 KB)

## Data Availability

All data used to support the findings of this study are directly available from the lead scientist (FPMO’H) upon request, without restrictions.

## Abbreviations

ALT: Alanine aminotransferase
APJ: Angiotensin II receptor-like protein J (apelin receptor)
AST: Aspartate aminotransferase
ELA: Exendin–linker–apelin
GLP-1: Glucagon-like peptide-1
HFF: High-fat-fed
RER: Respiratory exchange ratio

## References

[1] Huang Q, Li Y, Yu M et al (2025) Global burden and risk factors of type 2 diabetes mellitus from 1990 to 2021, with forecasts to 2050. Front Endocrinol 16:1538143. 10.3389/fendo.2025.1538143 PMC12390814 40895618

[2] Yau B, Ghislain J, Kebede MA, Hughes J, Poitout V (2025) The role of the beta cell in type 2 diabetes: new findings from the last 5 years. Diabetologia 68(10):2092–2103. 10.1007/s00125-025-06499-z PMC12423227 40768044

[3] Nauck MA, Quast DR, Wefers J, Meier JJ (2021) GLP-1 receptor agonists in the treatment of type 2 diabetes – state-of-the-art. Mol Metab 46:101102. 10.1016/j.molmet.2020.101102 PMC8085572 33068776

[4] Jastreboff AM, Aronne LJ, Ahmad NN et al (2022) Tirzepatide once weekly for the treatment of obesity. N Engl J Med 387(3):205–16. 10.1056/nejmoa2206038 35658024

[5] Filippatos TD, Panagiotopoulou TV, Elisaf MS (2015) Adverse effects of GLP-1 receptor agonists. Rev Diabet Stud 11(3):202. 10.1900/rds.2014.11.202 PMC5397288 26177483

[6] Gasoyan H, Butsch WS, Casacchia NJ et al (2025) Reasons for discontinuation of obesity pharmacotherapy with semaglutide or tirzepatide in clinical practice. Obesity 33(12):2296–303. 10.1002/oby.70058 PMC12636059 41039650

[7] Kang YM, Punov V, Lim S, Nauck MA (2025) Comparative efficacy and tolerability of currently approved incretin mimetics: a systematic analysis of placebo-controlled clinical trials. Diabetes Obes Metab 27(7):3736–46. 10.1111/dom.16398 PMC12146062 40212008

[8] El-Tanani M, Rabbani SA, El-Tanani Y, Matalka II, Khalil IA (2025) Bridging the gap: From petri dish to patient – advancements in translational drug discovery. Heliyon 11(1):e41317. 10.1016/j.heliyon.2024.e41317 PMC11730937 39811269

[9] Sridhar A, Palmer ES, Craig SL, Krason-Kidzinska E, Irwin N, O’Harte FP (2026) Unimolecular GLP-1/apelin hybrid peptides cause prominent appetite suppression, as well as enhancing insulin secretion, beta-cell survival and glycaemic regulation. Diabetes Obes Metab 28(6):4706–4717. 10.1111/dom.70647 PMC13146130 41834752

[10] Marzook A, Tomas A, Jones B (2021) The interplay of glucagon-like peptide-1 receptor trafficking and signalling in pancreatic beta cells. Front Endocrinol 12:678055. 10.3389/fendo.2021.678055 PMC8143046 34040588

[11] Mehri K, Hamidian G, Zavvari Oskuye Z, Nayebirad S, Farajdokht F (2023) The role of apelinergic system in metabolism and reproductive system in normal and pathological conditions: an overview. Front Endocrinol 14:1193150. 10.3389/fendo.2023.1193150 PMC10324965 37424869

[12] Tanday N, Flatt PR, Irwin N (2022) Metabolic responses and benefits of glucagon-like peptide-1 (GLP-1) receptor ligands. Br J Pharmacol 179(4):526–41. 10.1111/bph.15485 PMC8820187 33822370

[13] Palmer ES, Irwin N, O’Harte FP (2022) Potential therapeutic role for apelin and related peptides in diabetes: an update. Clin Med Insights Endocrinol Diabetes 15:11795514221074680. 10.1177/11795514221074679 PMC8844737 35177945

[14] Alfonso DM (2026) Apelin/APJ signalling as a potential complementary target for glucagon-like peptide-1 receptor agonist therapy in heart failure. Card Fail Rev 12:e03. 10.15420/cfr.2025.54 PMC12983138 41837114

[15] Hasebe M, Su CY, Keidai Y, et al (2025) Cardiovascular risk reduction with glucagon-like peptide-1 receptor agonists is proportional to HbA1c lowering in type 2 diabetes: an updated meta-regression analysis incorporating FLOW and SOUL trials. Diabetes Obes Metab 27(12):7210–7220. 10.1111/dom.70121 PMC12587236 40926380

[16] Przewlocka-Kosmala M, Kotwica T, Mysiak A, Kosmala W (2011) Reduced circulating apelin in essential hypertension and its association with cardiac dysfunction. J Hypertens 29(5):971–9. 10.1097/HJH.0b013e328344da76 21346619

[17] Irwin N, Gault VA, Green BD et al (2005) Antidiabetic potential of two novel fatty acid derivatised, N-terminally modified analogues of glucose-dependent insulinotropic polypeptide (GIP): N-AcGIP (LysPAL 16) and N-AcGIP (LysPAL 37). Biol Chem 386(7):679–687. 10.1515/bc.2005.079 16207089

[18] O’Harte FP, Parthsarathy V, Hogg C, Flatt PR (2018) Long-term treatment with acylated analogues of apelin-13 amide ameliorates diabetes and improves lipid profile of high-fat fed mice. PLoS One 13(8):e0202350. 10.1371/journal.pone.0202350 PMC6114795 30157220

[19] Flatt PR, Bailey CJ (1982) Plasma glucose and insulin responses to glucagon and arginine in Aston *ob*/*ob* mice: evidence for a selective defect in glucose-mediated insulin release. Horm Metab Res 14(03):127–30. 10.1055/s-2007-1018945 7040198

[20] Khan D, Vasu S, Moffett RC, Irwin N (1861) Flatt PR (2017) Influence of neuropeptide Y and pancreatic polypeptide on islet function and beta-cell survival. Biochim Biophys Acta Gen Subj 4:749–58. 10.1016/j.bbagen.2017.01.005 28069397

[21] Sridhar A, Khan D, Flatt PR, Irwin N, Moffett RC (2023) PYY (3–36) protects against high fat feeding induced changes of pancreatic islet and intestinal hormone content and morphometry. Biochim Biophys Acta Gen Subj 1867(6):130359. 10.1016/j.bbagen.2023.130359 37001706

[22] Halawi H, Khemani D, Eckert D (2017) Effects of liraglutide on weight, satiation, and gastric functions in obesity: a randomised, placebo-controlled pilot trial. Lancet Gastroenterol Hepatol 2(12):890–9. 10.1016/s2468-1253(17)30285-6 28958851

[23] Nemati M, Zardooz H, Rostamkhani F, Abadi A, Foroughi F (2017) High-fat diet effects on metabolic responses to chronic stress. Arch Physiol Biochem 123(3):182–91. 10.1080/13813455.2017.1295083 28276709

[24] McCrimmon RJ, Catarig AM, Frias JP et al (2020) Effects of once-weekly semaglutide vs once-daily canagliflozin on body composition in type 2 diabetes: a substudy of the SUSTAIN 8 randomised controlled clinical trial. Diabetologia 63(3):473–85. 10.1007/s00125-019-05065-8 PMC6997246 31897524

[25] Neeland IJ, Linge J, Birkenfeld AL (2024) Changes in lean body mass with glucagon-like peptide-1-based therapies and mitigation strategies. Diabetes Obes Metab 26(4):16–27. 10.1111/dom.15728 38937282

[26] Langer HT, Gilmore NK, Hayden CM et al (2026) Weight loss with GLP-1 medicines does not result in a disproportionate loss of muscle mass or function in obese mice and humans. Cell Rep Med 7(3):102665. 10.1016/j.xcrm.2026.102665 PMC13006392 41850248

[27] Karakasis P, Patoulias D, Fragakis N, Mantzoros CS (2025) Effect of glucagon-like peptide-1 receptor agonists and co-agonists on body composition: systematic review and network meta-analysis. Metabolism 164:156113. 10.1016/j.metabol.2024.156113 39719170

[28] Jensterle M, Janež A (2026) Rethinking muscle health assessment in GLP-1-based therapies for obesity: beyond lean mass. Diabetes Obes Metab 28(6):4428–4431. 10.1111/dom.70649 41804837

[29] Vinel C, Lukjanenko L, Batut A et al (2018) The exerkine apelin reverses age-associated sarcopenia. Nat Med 24(9):1360–71. 10.1038/s41591-018-0131-6 30061698

[30] Irwin N, Montgomery IA, Flatt PR (2013) Comparison of the metabolic effects of sustained CCK1 receptor activation alone and in combination with upregulated leptin signalling in high-fat-fed mice. Diabetologia 56(6):1425–35. 10.1007/s00125-013-2878-0 23462797

[31] Folmer V, Soares JC, Gabriel D, Rocha JB (2003) A high fat diet inhibits δ-aminolevulinate dehydratase and increases lipid peroxidation in mice (*Mus musculus*). J Nutr 133(7):2165–70. 10.1093/jn/133.7.2165 12840172

[32] Kuti D, Winkler Z, Horváth K et al (2022) The metabolic stress response: adaptation to acute-, repeated-and chronic challenges in mice. iScience 25(8):104693. 10.1016/j.isci.2022.104693 PMC9307515 35880047

[33] Attané C, Foussal C, Le Gonidec S et al (2012) Apelin treatment increases complete fatty acid oxidation, mitochondrial oxidative capacity, and biogenesis in muscle of insulin-resistant mice. Diabetes 61(2):310–20. 10.2337/db11-0100 PMC3266414 22210322

[34] Wei Q, Li L, Chen JA, Wang SH, Sun ZL (2015) Exendin-4 improves thermogenic capacity by regulating fat metabolism on brown adipose tissue in mice with diet-induced obesity. Ann Clin Lab Sci 45(2):158–65 25887869

[35] Zitting KM, Vujovic N, Yuan RK et al (2018) Human resting energy expenditure varies with circadian phase. Curr Biol 28(22):3685–90. 10.1016/j.cub.2018.10.005 PMC6300153 30416064

[36] Dickson SL, Shirazi RH, Hansson C, Bergquist F, Nissbrandt H, Skibicka KP (2012) The glucagon-like peptide 1 (GLP-1) analogue, exendin-4, decreases the rewarding value of food: a new role for mesolimbic GLP-1 receptors. J Neurosci 32(14):4812–20. 10.1523/jneurosci.6326-11.2012 PMC6620919 22492036

[37] Alhazmi A, le Roux CW (2026) Do no harm: managing nausea and vomiting in GLP-1 based obesity therapies. Front Endocrinol 17:1788698. 10.3389/fendo.2026.1788698 PMC12992036 41852477

[38] Leech CA, Dzhura I, Chepurny OG et al (2011) Molecular physiology of glucagon-like peptide-1 insulin secretagogue action in pancreatic β cells. Prog Biophys Mol Biol 107(2):236–47. 10.1016/j.pbiomolbio.2011.07.005 PMC3200499 21782840

[39] Hu G, Wang Z, Zhang R, Sun W, Chen X (2021) The role of apelin/apelin receptor in energy metabolism and water homeostasis: a comprehensive narrative review. Front Physiol 12:632886. 10.3389/fphys.2021.632886 PMC7928310 33679444

[40] Diouri O, Cigler M, Vettoretti M et al (2021) Hypoglycaemia detection and prediction techniques: a systematic review on the latest developments. Diabetes Metab Res Rev 37(7):e3449. 10.1002/dmrr.3449 PMC8519027 33763974

[41] Goedeke L, Strober JW, Suh R et al (2024) High-fat-diet-induced hepatic insulin resistance per se attenuates murine de novo lipogenesis. iScience 27(11):111175. 10.1016/j.isci.2024.111175 PMC11550620 39524330

[42] Yue P, Jin H, Aillaud M et al (2010) Apelin is necessary for the maintenance of insulin sensitivity. Am J Physiol Endocrinol Metab 298(1):E59–E67. 10.1152/ajpendo.00385.2009 PMC2806109 19861585

[43] Jin T, Weng J (2016) Hepatic functions of GLP-1 and its based drugs: current disputes and perspectives. Am J Physiol Endocrinol Metab 311(3):E620–E627. 10.1152/ajpendo.00069.2016 27507553

[44] Targher G, Corey KE, Byrne CD, Roden M (2021) The complex link between NAFLD and type 2 diabetes mellitus – mechanisms and treatments. Nat Rev Gastroenterol Hepatol 18(9):599–612. 10.1038/s41575-021-00448-y 33972770

[45] Drucker DJ (2003) Glucagon-like peptide-1 and the islet β-cell: augmentation of cell proliferation and inhibition of apoptosis. Endocrinology 144(12):5145–8. 10.1210/en.2003-1147 14645210

[46] Han S, Englander EW, Gomez GA et al (2015) Pancreatic islet APJ deletion reduces islet density and glucose tolerance in mice. Endocrinology 156(7):2451–60. 10.1210/en.2014-1631 25965959

[47] Cerf ME (2015) High fat programming of beta cell compensation, exhaustion, death and dysfunction. Pediatr Diabetes 16(2):71–8. 10.1111/pedi.12137 25682938

[48] Lee DK, Ferguson SS, George SR, O’Dowd BF (2010) The fate of the internalized apelin receptor is determined by different isoforms of apelin mediating differential interaction with beta-arrestin. Biochem Biophys Res Commun 395(2):185–9. 10.1016/j.bbrc.2010.03.151 20353754

[49] Tanday N, Tarasov AI, Moffett RC, Flatt PR, Irwin N (2024) Pancreatic islet cell plasticity: Pathogenic or therapeutically exploitable? Diabetes Obes Metab 26(1):16–31. 10.1111/dom.15300 37845573

[50] Tanday N, Irwin N, Moffett RC, Flatt PR, O’Harte FP (2020) Beneficial actions of a long-acting apelin analogue in diabetes are related to positive effects on islet cell turnover and transdifferentiation. Diabetes Obes Metab 22(12):2468–78. 10.1111/dom.14177 32844576

[51] Tanday N, Flatt PR, Irwin N, Moffett RC (2020) Liraglutide and sitagliptin counter beta-to alpha-cell transdifferentiation in diabetes. J Endocrinol 245(1):53–64. 10.1530/joe-19-0451 31977315

[52] Strutt B, Szlapinski S, Gnaneswaran T, et al (2021) Ontology of the apelinergic system in mouse pancreas during pregnancy and relationship with β-cell mass. Sci Rep 11(1):15475. 10.1038/s41598-021-94725-0 PMC8322410 34326390

[53] Dubey V, Tanday N, Ali A, et al (2025) Effect of multiparity on pregnancy-induced islet adaptation and cellular transdifferentiation. Clin Med Insights Endocrinol Diabetes 18:11795514251386124. 10.1177/11795514251386124 PMC12536105 41122679

