# Supplementary material for: Unimolecular GLP-1/APJ receptor co-agonism improves metabolism and dyslipidaemia as well as normalising hepatic triglyceride and aminotransferase elevation in high-fat-fed mice
Source: Diabetologia. 2026 Sep 18;69(12):3654–66. doi: 10.1007/s00125-026-06863-7 (PMC13714080; doi:10.1007/s00125-026-06863-7)
Supplement: Supplementary file 1 [file 125_2026_6863_MOESM1_ESM.pdf]

Unimolecular GLP 1/APJ receptor co-agonism improves metabolism and dyslipidaemia as well as hepatic triglyceride and aminotransferase elevation in high-fat-fed mice

ESM Methods

Animals and treatment schedules

Male C57BL/6 mice (12 weeks of age, Envigo, UK) were housed individually in an air-conditioned room at 22 ± 2°C with 12 h light and dark cycle with *ad libitum* access to drinking water. Mice were maintained on a high fat diet (45% fat, 20% protein and 35% carbohydrate; D08112601i, Research Diets Inc., New Brunswick, NJ, USA) or standard rodent diet (D12450J, 10% fat, 30% protein and 60% carbohydrate (Research Diets, Inc.) as appropriate, for 14 weeks. By this point, all high fat fed (HFF) mice had significantly elevated body weight when compared to mice fed standard rodent maintenance diet. No adverse effects were noted following injection of all peptides as detailed within the main manuscript.

Metabolic analyses

Indirect calorimetry was assessed using an Oxymax Comprehensive Laboratory Animal Monitoring System (CLAMS), with 18 h acclimation prior to recordings (Columbus Instruments, Columbus, OH, USA). Following the acclimatisation period, respiratory exchange ratio (RER), energy expenditure and locomotor activity were assessed. Total body fat and lean masses were measured by dual-energy X-ray absorptiometry (DEXA) scanning (Piximus Densitometer, Inside Outside Sales, Madison WI, USA). All animal procedures were performed in compliance with the UK Animal (Scientific Procedures) Act 1986, covered under a UK Home Office Animal project license (UK Home Office license certificate PPL2902 approved on 26 April 2021), and approved by the Ulster Animal Welfare and Ethical Review Body (AWERB) committee.

Biochemical analyses

For plasma insulin analyses by radioimmunoassay (RIA), blood was also collected in heparin/fluoride coated microcentrifuge tubes (Sarstedt, Nümbrecht, Germany) and centrifuged at 1500 *g* for 15 min at 4°C in a microcentrifuge to extract plasma. Circulating triglycerides, high-density lipoprotein (HDL)-cholesterol and low-density lipoprotein (LDL)-cholesterol, as well as the liver enzymes alanine aminotransferase (ALT) and aspartate aminotransferase (AST), were measured using a Hitachi Automated Analyser 912 (Boehringer Ingelheim, Mannheim, Germany). Hepatic triglycerides were extracted in ethanol (1 g tissue:9 ml ethanol), generating homogenates of equivalent concentrations. Triglycerides were then quantified by colorimetric analysis, following the manufacturer's protocol (Triglyceride (TG) Colorimetric Assay Kit, EEA028, Invitrogen, ThermoFisher Scientific, Altrincham, Cheshire, UK).

Pancreatic islet immunohistochemistry

At termination, pancreatic tissue was excised and immediately fixed in 4% paraformaldehyde for pancreatic islet histology assessment. Islet morphology was then examined by immunohistochemical staining for insulin (1:400; ab6995; Abcam) or glucagon (1:1000; ab92517; Abcam). Image analysis was conducted using Cell^F image analysis software (Olympus Soft Imaging Solutions, GmbH, Münster, Germany). To assess islet morphology, areas of insulin- and glucagon-positive staining were quantified using a 'closed polygon' tool and expressed as islet/beta-/alpha-cell areas in μm^2, with islet area being considered as the total of insulin- and glucagon-positive stained cells. Beta-cell proliferation and apoptosis were investigated by co-staining of insulin with Ki-67 (1:400; ab15580, Abcam) or terminal deoxynucleotidyl transferase-mediated dUTP nick end labeling (TUNEL) (*in situ* cell death

kit, Fluorescein; Roche Diagnostics, West Sussex, UK). For quantification, the number of insulin-positive cells co-expressing Ki-67 or TUNEL, as appropriate, were counted using ImageJ software, with >80 islets analysed per treatment group. For all islet immunohistochemistry studies, following incubation with primary antibodies, relevant secondary antibodies were employed, including Alexa Fluor594 goat anti-mouse IgG or Alexa Fluor488 goat anti-rabbit (1:400; Invitrogen, ThermoFisher Scientific, Altrincham, Cheshire, UK).
